# Oral disease burden of dentate older adults living in long-term care facilities: FINORAL study

**DOI:** 10.1186/s12903-021-01984-4

**Published:** 2021-12-07

**Authors:** Lina Julkunen, Kaija Hiltunen, Hannu Kautiainen, Riitta K. T. Saarela, Kaisu H. Pitkälä, Päivi Mäntylä

**Affiliations:** 1 Oral and Maxillofacial Diseases Outpatient Clinic, Helsinki University Hospital, Helsinki, Finland; 2 Department of Oral and Maxillofacial Diseases, University of Helsinki, Helsinki, Finland; 3 Primary Health Care Unit, Kuopio University Hospital, Kuopio, Finland; 4 Folkhälsan Research Center, Helsinki, Finland; 5 Department of Social Services and Health Care, Oral Health Care, City of Helsinki, Finland; 6 Department of General Practice, University of Helsinki, Helsinki, Finland; 7 Unit of Primary Health Care, Helsinki University Hospital, Helsinki, Finland; 8 Institute of Dentistry, University of Eastern Finland, Yliopistonranta 1 B, P. O. Box 1627, 70211 Kuopio, Finland; 9 Oral and Maxillofacial Diseases, Kuopio University Hospital, Kuopio, Finland

**Keywords:** Older adults, Oral diseases, Long-term care, Disease burden

## Abstract

**Background:**

A growing number of older adults have natural teeth and are at high risk of oral diseases, which are induced by oral bacterial accumulation and proceed unnoticed and quietly. Our aim was (1) to examine the association of oral disease burden (ODB) with health and functioning among dentate long-term care residents, and (2) to find easily detectable signs for nurses to identify residents’ poor oral health.

**Methods:**

In this cross-sectional observational study dentists examined 209 residents’ oral status, and nurses assessed residents for their functioning and nutrition in long-term care facilities in Helsinki, Finland. ODB was defined by asymptotic dental score (ADS). Six clinical signs of residents’ poor oral health were considered as potentially easy for nurses to detect: lesions on lips, teeth with increased mobility, lesions on oral mucosa, eating soft or pureed food, unclear speech, and needing assistance in eating. The association of these was tested with high ODB as outcome.

**Results:**

Participants were grouped according to their ADS scores: low (n = 39), moderate (n = 96) and high ODB (n = 74). ODB was linearly associated with coronary artery disease and poor cognitive and physical functioning: needing assistance in eating, poor ability to make contact, and unclear speech but not with other diseases including dementia or demographic characteristics. Furthermore, ODB was linearly associated with eating soft or pureed food. Of the six selected, easily detectable signs, having at least two positive signs gave 89% sensitivity to detecting high ODB.

**Conclusion:**

Poor oral health was common and ODB accumulated among residents with poor functioning. Nurses may use a few easily detectable signs to screen residents’ oral health when considering a resident’s need for consultation with an oral health professional.

## Background

A growing number of older adults have natural teeth and are at high risk for dental caries, periodontitis, associated systemic infections, and tooth loss [1]. Evidence indicates that poor oral health becomes more common with increasing age [2]. When sensory deficiencies, functional impairments, and cognitive decline become common along with age-related diseases, older people’s ability to take care of daily oral hygiene becomes difficult or even impossible. Increasing evidence shows that cardiovascular and respiratory diseases, as well as diabetes and Alzheimer’s disease, may have an association with oral inflammatory diseases [3–5]. Common oral infectious and inflammatory conditions such as periodontal diseases and caries proceed unnoticed and quietly, and pain is rare or only occurs when the disease is already at an advanced stage [6]. It is difficult to detect oral discomfort and pain and assess the mouth in frail older adults, especially if they suffer from cognitive decline [7]. Long-term care residents are practically all frail and immunologically compromised, and a rapid deterioration of remaining teeth because of caries or complete collapse of periodontal support is possible [8]. In previous studies, the prevalence of caries in long-term care facilities has ranged between 37 and 77% [9–13] and periodontal inflammation between 51 and 80% [9, 14]. Even though the oral health of long-term care residents is known to be poor, hardly any studies exist examining how nurses who frequently interact with residents could be trained to identify oral health care needs. In a study dating back to the last millennium nursing staff received very short (1–4 h) of training in oral health to identify the need for treatment, and the result was compared with dentist’s registrations [15]. Longer training led to better performance but fell far short of dentist’s findings. Training nurses to recognize specific signs associated with oral diseases would be useful to integrate into long-term care.

In this cross-sectional study, we examine the association of the burden of common oral diseases, which are induced by oral bacterial accumulation, with cognitive and physical functioning, physical findings, and diseases among dentate long-term care facility residents living in Helsinki, Finland. The study also aims to find out if easily detectable signs exist that could be used as markers of poor oral health and would be applicable for nursing home staff in identifying the persons in need of oral health care. The main hypothesis is that oral diseases cause stress, which is seen in overall health, and poor oral health can be identified by observing selected signs/markers.

## Methods

Population of the Finnish Oral health studies in Older Adults (FINORAL study) is a random subsample of participants of the nutrition study that included all older residents in capital area of Helsinki [16] living in long-term care (nursing homes and assisted living facilities) and who were 65 years or older (N = 3673). Data for the nutrition study were collected in March 2017. A sample of 550 of the participants in the nutrition study gave consent to participate the FINORAL study. Individuals needing prophylactic antibiotics (N = 47), and those who had major deficiencies or completely refused (N = 35) from the clinical examination were excluded. Of the participants, 75 were deceased between the end of nutritional and beginning of FINORAL study. Finally, a total of 393 individuals were included in the FINORAL study. Further, excluded from the current study were edentulous individuals (n = 100) and participants with incomplete information for asymptotic dental score (ADS) calculation and/or cases not able to be combined with the data of the concomitant nutrition study [15] for general health functioning information (n = 87) resulting in a study population of n = 209.

The participation was voluntary. Each participant or his/her closest proxy in cases where the resident concerned was not able to understand the content of the study, such as those having dementia, gave written informed consent. The City of Helsinki and the Ethics Committee of the Hospital District of Helsinki and Uusimaa approved the study protocol (HUS/2042/2016 and HUS/968/2017). This study adheres to the guidelines of the Declaration of Helsinki.

The registered nurse most familiar with the participants in each facility filled in a questionnaire concerning study participants’ demographic characteristics (age, sex, education). Residents’ diagnoses and use of medications were obtained from medical records. The Charlson comorbidity index was calculated as previously described [17]. Residents’ cognitive state was assessed by the Mini-mental state examination (MMSE) [18] and Clinical Dementia Rating (CDR) [19]. Residents were evaluated for their ability to move (independently with or without aids/needs assistance or unable to move) and eating (independently/needs assistance). Residents’ nutritional status was assessed with the Mini Nutritional Assessment (MNA) [20] and body mass index (BMI) was calculated. The nurses also clarified residents’ food consistency (normal/soft or pureed). The nurses were thoroughly trained to assess residents and fill in the questionnaires.

Two qualified and calibrated dentists conducted oral clinical examinations between September 2017 and January 2019. Examinations were carried out with normal set of sterile dental instrumentation and loupes (Merident Optergo MO Ultralight Flip-up) with an attached headlamp (Merident Optergo DeLight LED). Participants of the oral health study were lying in bed or sat in a chair during the oral examination.

The oral examination comprised visual examination of lips (healthy or chapped lips and/or cheilitis angularis); in the oral cavity inspection of oral mucosa (healthy mucosa, lesion not related with use of removable denture or removable denture-related lesion), clinical estimation of oral wetness [clinically normal (all surfaces of the mouth are moist, no sticking of the mirror on oral mucosa) or signs of reduced salivation / dry mouth (mirror sticks to buccal mucosa or tongue, frothy saliva, glassy appearance of oral palate lobulated/fissured tongue)], modified from Osailan et al. [21], number of natural teeth including root remnants, and use of removable denture; bacterial plaque accumulation (plaque index, PI, according to the modified Silness and Löe index, values 0 no plaque to 4 whole tooth covered with plaque) [22], level of gingival inflammation (gingival index, GI, values 0 no inflammation to 3 severe inflammation) [22], both registered as the highest score for each tooth and calculated as the mean value for the whole dentition; visually assessed open caries lesions of the tooth crown and root caries lesions; pocket probing depth (PPD) measurements from 4 sites (mid- and distobuccal, mid- and mesiolingual) and registered as the deepest PPD for each tooth (< 4 mm, 4–5 mm, ≥ 6 mm); bleeding on probing (BOP, yes/no for each tooth), and tooth mobility assessed in static mode (horizontal mobility for each tooth with a handle of a metal instrument and one finger, and axially with an instrument; registered either no distinguishable sign of movement greater than physiologic or greater than physiologic) [23]. Further, the percentage of teeth with plaque was calculated for all study participants. Dentists evaluated study participants’ ability to make contact (ability to contact the researcher, respond, and seek to assist in progress of the examination, good/weakened), and clarity of speech (clear/unclear or unable to speak).

Oral disease burden (ODB) was described by asymptotic dental score (ADS) which sums up oral pathologies [24]. The ADS was modified for the purpose of this study to comprise clinical oral examination variables. Included variables were 1. dental caries or one edentulous jaw in line with Janket et al. [24] (values: 0 = no caries, 1 = 1–3 caries lesions, 2 = 4–7 caries lesions or one edentulous jaw, 3 =  ≥ 8 caries lesions); 2. gingivitis (GI ≥ 1 and/or BOP ≥ 20%; values: 0 no, 1 yes) in similar way as Janket et al. [24] who observed if gingival tissue exhibited overt signs of inflammation (erythema, bleeding, and papillary or generalized swelling); 3. root remnants in line with Janket et al. [24] (values: 0 = no root remnants, 1 = one root remnant, 2 = two or more root remnants); and 4. the number of teeth with deepened periodontal pockets as an indication of inflamed gingival surface [number of teeth with PPD 4–5 mm plus weighted (multiplied by two) number of teeth with PPD ≥ 6 mm] [25]; values: 0 = no pockets, 1 = 1–3 pockets, 2 = 4–10 pockets, 3 = 11 or more pockets), while Janket et al. [24] measured a proxy for periodontal disease (yes/no) by using the community periodontal index of treatment need (CPITN) (if at least 2 sextants were recorded as having CPITN ≥ 3 signifying that sextant had periodontal pocket depth ≥ 3.5 mm). No X-rays were taken and thus no radiologic findings could be included in the scoring. The final ADS score of each participant varied from 0 to 9 which as a continuous value showed a normal distribution. Participants were divided into three ODB groups: no or low (ADS 0–2), moderate (ADS 3–4), and high (ADS 5–9) oral disease burden (mentioned below as ADS low, ADS moderate, ADS high).

### Statistics

The categorical variables were described as numbers and percentages (%), and the continuous variables as means and standard deviations (SDs). The linearity across the three-level groups of ADS were evaluated using the Cochran-Armitage test (chi-square test for trend), logistic models and analysis of variance with an appropriate contrast (orthogonal). Prediction of ODB (ADS high as outcome) with individual oral signs score-6 analysis (lesions on lips, teeth with increased mobility, lesions on oral mucosa, eating soft or pureed diet, unclear speech, and eating as assisted; each yes/no) items and as a sum score were evaluated using AUC (area under curve), sensitivity, specificity, positive and negative predictive values, and likelihood ratio; 95% confidence intervals were obtained by bias corrected bootstrapping (5000 replications). An exploratory factor analysis with the iterated principal-factor method for factoring and promax-rotated factor loadings on polychoric correlation matrix was performed to identify related items in the oral signs score-6. Promax rotation is an alternative nonorthogonal rotation method. The strategies used to extract the number of factors were: the Kaiser criteria, which determine that components with eigenvalues lower than one should be excluded, and the screen test of Cattell criteria. Correlation coefficients with 95% confidence interval (CI) were calculated by using the Spearman method. The normality of variables was evaluated graphically and by using the Shapiro–Wilk W test. Stata 16.0 (StataCorp LP, College Station, TX, USA) was used for the statistical analyses.

## Results

The study population comprising older adults in long-term care facilities was divided into three ODB groups according to the clinical asymptotic dental score: ADS low (n = 39), ADS moderate (n = 96), and ADS high (n = 74). Table 1 depicts the variables included in the ADS score calculation and the distribution of values in each ADS group.

**Table 1:** Variables included in the asymptotic dental score (ADS) calculation for ADS groups low to high

	ADS low N = 39	ADS moderate N = 96	ADS high N = 74	*p* value*
Root remnants, mean (SD)	0.1 (0.3)	1.1 (2.4)	2.4 (2.6)	< 0.001
Teeth with PPD 4–5 mm, mean (SD)	0.7 (1)	2.7 (4)	6.7 (5.2)	< 0.001
Teeth with PPD ≥ 6 mm, mean (SD)	0.1 (0.3)	0.4 (1.4)	1.5 (2.3)	< 0.001
% of BOP positive teeth, mean (SD)	64 (40)	87 (28)	87 (28)	< 0.001
Gingival index, mean (SD)	1.0 (0.7)	1.8 (0.8)	1.9 (0.7)	< 0.001
Teeth with root caries, mean (SD)	0.3 (0.6)	0.7 (1.1)	2.7 (3.1)	< 0.001
Teeth with open caries lesion, mean (SD)	0.1 (0.4)	0.4 (1)	1.0 (1.3)	< 0.001
Edentulous jaw n (%)	6 (15)	34 (37)	19 (26)	0.46

*PPD* pocket probing depth, *BOP* bleeding on probing

*p for linearity

The participants’ demographic, health and functional characteristics are presented in Table 2. The participants’ mean age was 82 years and 72% of them were females. About 68% suffered from dementia. According to CDR, the severity of symptoms of dementia was at moderate to severe stages for 74% of participants. In addition, the declining trend of MMSE points from ADS low (low ODB) to ADS high (high ODB) reached statistical significance.

**Table 2:** Characteristics of study participants stratified by oral disease burden (ODB) defined by asymptotic dental score (ADS)

	ADS low n = 39	ADS moderate n = 96	ADS high n = 74	*p* value*
*Demographic characteristics*				
Age, mean (SD)	81 (9)	82 (8)	84 (9)	0.12
Gender female, n (%)	29 (74)	62 (65)	60 (81)	0.22
Education ≤ 8 years, n (%)	16 (42)	34 (39)	23 (34)	0.41
*Health and diseases*				
Charlson comorbidity index, mean (SD)	2.0 (1.5)	1.9 (1.2)	2.1 (1.3)	0.78
Coronary artery disease, n (%)	3 (8)	9 (9)	15 (20)	0.032
Myocardial infarction, n (%)	3 (8)	1 (1)	5 (7)	0.81
Stroke, n (%)	10 (26)	23 (24)	23 (31)	0.43
Dementia, n (%)	24 (62)	65 (68)	54 (73)	0.21
Regular medications, mean (SD)	9.0 (3.4)	9.4 (3.8)	8.6 (3.7)	0.39
*Functioning*				
Ability to move, n (%): independently with or without aids	22(56)	33(35)	27(38)	0.11
Eating, n (%): needs assistance	13 (33)	44 (43)	39 (53)	0.035
Daily oral hygiene, n (%): needs assistance	12 (32)	31 (33)	29 (40)	0.33
Dementia stage CDR, n (%)				0.26
Mild or very mild	11 (29)	28 (31)	14 (19)	
Moderate	9 (24)	26 (29)	21 (29)	
Severe	18 (47)	36 (40)	38 (52)	
MMSE, mean (SD)	16.5 (7.8)	14.1 (7.6)	13.6 (6.8)	0.084
Ability to make contact, n (%): weakened	17 (44)	57 (61)	48 (66)	0.033
Unclear or no speech, n (%)	9 (23)	40 (43)	36 (49)	0.012
*Nutrition*				
BMI, mean (SD)	26.5 (6.3)	26.1 (4.7)	25.7 (5.2)	0.47
Consistency of food, n (%): soft or pureed	5 (13)	22 (23)	25 (34)	0.012
MNA, mean (SD)	21(4)	21(3)	20(3)	0.25

*MMSE* mini-mental state examination, *BMI* body mass index, *MNA* mini nutritional assessment

*p for linearity

Worsening ODB according to ADS was linearly associated with coronary artery disease (CAD) but not with dementia or other disorders. ODB was linearly associated with functional variables: ability to make contact and clarity of speech. In addition, those with high ODB more often needed assistance in eating and received soft or pureed food (Table 2).

Among general oral findings (not included in the ADS calculation) a linear association with ODB was found for lesions on lips (cheilitis angularis and/or chapped lips), lesions on oral mucosa, teeth with increased mobility, and plaque index value (Table 3). However, there was no association between ODB according to ADS and receiving assistance in daily oral hygiene, share (%) of teeth with bacterial plaque, use of dentures or oral mucosal wetness. Mucosal wetness was regarded as clinically normal in 31% of participants in ADS groups low and moderate and 18% in ADS high.

**Table 3:** General oral findings (not included in the asymptotic dental score, ADS, calculation)

	ADS low n = 39	ADS moderate n = 96	ADS high n = 74	*p* value*
*Skin, lips, and oral mucosa*				
Lesions on lips (chapped lips and/or cheilitis angularis), n (%)	3 (8)	29 (30)	44 (59)	< 0.001
Lesions on oral mucosa, n (%)	1 (3)	19 (20)	22 (30)	< 0.001
Mucosal wetness, clinically, n (%) normal	12 (31)	29 (31)	13 (18)	0.10
*Teeth and dentures*				
Teeth^a^, mean (SD)	16.7 (7.2)	12.9 (8.8)	13.3 (7.7)	0.078
Teeth with increased mobility, mean (SD)	0.7 (2.1)	1.0 (2.1)	3.1 (3.8)	< 0.001
Plaque index, mean (SD)	2.0 (0.9)	2.5 (1.0)	2.6 (0.8)	0.008
Percentage of teeth with plaque, mean (SD)	94 (19)	96 (17)	95 (17)	0.62
Removable denture in use, n (%)	8 (22)	22 (23)	14 (19)	0.66

^a^Root remnants not included

*p for linearity

**Fig. 1:**
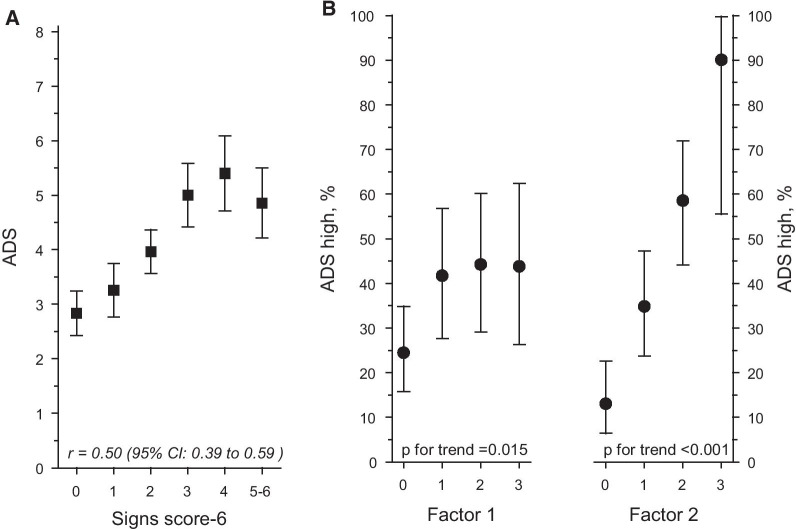
**A** Relationship between the number of signs score-6 items and asymptotic dental score (ADS). ADS asymptotic dental score (mean with 95% CI); Spearman correlation coefficient (r) with 95% CI. **B** Signs score-6 loaded into two factors. Relationship between number of signs and ADS high (high ODB). Factor 1, oral function: eating soft or pureed food, inarticulate speaking, needing assistance in eating; Factor 2, oral diseases: lesions on lips, teeth with increased mobility, lesions on oral mucosa; ADS asymptotic dental score high (mean with 95% CI)

Of study participants 35% had ADS high (high ODB). We further analyzed whether certain signs could be used to identify residents with the worst ODB (ADS high). Variables included were lesions on lips, teeth with increased mobility, lesions on oral mucosa, eating soft or pureed food, clarity of speech, and needing assistance in eating. We constructed an oral signs score-6 in which each positive sign gave one point. This score had high correlation with ADS score (Fig. 1A). In an exploratory factor analysis these six signs were loaded into two main factors (Factor 1, oral function: eating soft or pureed food, clarity of speech, needing assistance in eating; Factor 2, oral diseases: lesions on lips, mobile teeth, lesions on oral mucosa) (Table 4). These factors explained 76% of the total variance of the entire study population. Correlation between factors 1 and 2 and ADS high are shown in Fig. 1B.

**Table 4:** Exploratory factor analysis with factor loadings of the oral sings score-6 items

	Factor 1 Oral function	Factor 2 Oral diseases
Consistency of food soft or pureed	0.79	
Unclear speech	0.87	
Needs assistance in eating	0.91	
Lesions on lips		0.75
Teeth with increased mobility		0.77
Lesions on oral mucosa		0.55

Coefficients with values < 0.50 not shown. Factors explained 76% of the total variance

**Fig. 2:**
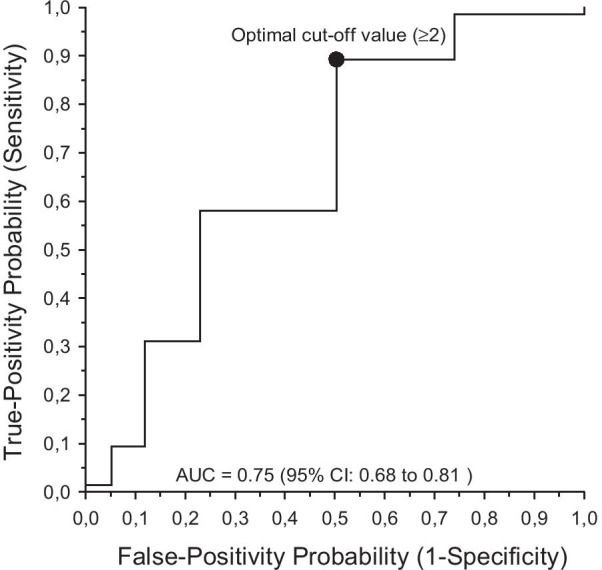
Receiver operating characteristic (ROC) curve built for signs score-6 items. The best cut-off point for 2 signs or more provided sensitivity 89% (95% CI 0.80–0.95) for detecting ADS high (high ODB). *AUC* area under curve, *95% CI* confidence interval

Table 5 shows summary statistics for diagnostic tests for individual oral signs score-6 items to find ADS high. Diagnostic value of individual signs was relatively low with area under curve (AUC) values ranging from 0.56 to 0.68 and positive predictive values from 42 to 58%. Highest sensitivity (64%) was found for teeth with increased mobility and highest specificity for lesions on oral mucosa (85%). In general, specificity values were higher for all items compared to sensitivities. We built up a receiver operating characteristic (ROC) curve analysis for combined use of these signs (Fig. 2). It showed the best cut-off point for two signs or more to provide 89% sensitivity (95% CI 0.80–0.95) for detecting ADS high (high ODB).

**Table 5:** Summary statistics for diagnostic values of individual signs score-6 items to find asymptotic dental score (ADS) high (high oral disease burden, ODB)

	AUC^a^	Sensitivity	Specificity	Positive predictive value	Likelihood ratio of a positive test
	% (95% CI)				
Consistency of food soft or pureed	0.57 (0.50–0.63)	34 (23–46)	80 (72–86)	48 (34–62)	1.69 (1.06–2.69)
Unclear speech	0.56 (0.49–0.63)	49 (37–61)	64 (55–72)	42 (32–54)	1.34 (0.97–1.85)
Needs assistance in eating	0.56 (0.49–0.63)	53 (41–64)	60 (51–68)	42 (32–53)	1.32 (0.98–1.78)
Lesions on lips	0.68 (0.61–0.75)	59 (47–71)	76 (68–83)	58 (46–69)	2.51 (1.76–3.58)
Teeth with increased mobility	0.67 (0.60–0.74)	64 (52–74)	70 (62–78)	54 (43–65)	2.14 (1.57–2.93)
Lesions on oral mucosa	0.57 (0.51–0.64)	30 (20–41)	85 (78–91)	52 (36–68)	2.01 (1.18–3.43)

^a^Area under ROC

## Discussion

In this cross-sectional study, we determined the total burden of clinically detectable oral biofilm-induced diseases among dentate older adults living in long-term care facilities, and its association with their health and functioning. The highest level of ODB associated with poor functioning rather than individual diseases or multi-morbidity. In addition, we found that the individuals with the worst oral health might be identified by looking at a variety of readily observable clinical signs such as having lip lesions, teeth with increased mobility, lesions on oral mucosa, unclear speech, eating soft or pureed food and needing assistance in eating; individually assessed to get a preliminary idea and in combination to confirm the situation. These signs could be used by nurses as markers to detect oral problems and identify the need for consultation with an oral health professional.

The strength of the study is a relatively large sample most vulnerable older adults with 67% of participants being frail according to frailty index [26], who have retained at least some of their own natural teeth and whose oral status was comprehensively examined by dentists. As a result, we have been able to demonstrate the scale and extent of oral problems. In addition, this study is, to the best of our knowledge, the first to use a score which brings together several clinically detectable oral diseases and/or their manifestations to assess their associations with general health and functioning among older adults living in long-term care facilities. For this purpose, we used the earlier validated Asymptotic Dental Score (ADS) [24] modified for our purposes.

The main limitation is the cross-sectional design of the study, due to which it was not possible to detect cause-and-effect relationships. Another limitation is the relatively low power of the study due to the number of older adults with a low level of ODB. It may be the reason why only large differences between study groups could be detected. We could not take X-rays, and as a result it is likely that a significant proportion of oral sources of inflammation, such as periapical infections or any other lesions affecting jawbones, went undetected. In addition, the interval between nutrition study and oral examination was more than 1 year for about one third of the participants, which may affect the relationship between the oral and general/nutritional variables.

The oral health of older people living in institutional care has been found to be at an unacceptable level [9–14, 27, 28]. Our result is in line with earlier studies, since only 19% of examined older adults had a low level of ODB. In our sample, ODB was expressed by ADS, which includes clinical findings related to caries and periodontal diseases and their final consequences: root remnants and loss of multiple teeth. Caries and periodontitis are the main causes of tooth loss globally [29]. The main initiator of these common oral diseases is the buildup of bacterial plaque. Bacterial accumulations on the surfaces of teeth were measured as plaque index, and it increased with the increasing ODB.

Symptoms of these most common oral diseases are usually non-existent or minor and go unnoticed by individuals, and only at advanced stages cause subjective sensations or pain. For this reason, they are difficult to detect at early or even at rather advanced stages other than by a professional oral examination. Nurses experience challenges in recognizing oral diseases [30, 31]. Inflammation caused by these diseases increases systemic inflammatory load, which may be detrimental to the frail and multimorbid older adults living in long-term care residencies [32].

In line with prior studies, older adults’ oral health showed a significant association with functioning [33, 34]. In our study, needing assistance in eating, unclear speech, and poor ability to make contact were associated with ODB severity. However, contrary to previous studies, ODB was not associated with mobility [35, 36]. Furthermore, in line with prior studies, ODB was associated with cognitive decline [37, 38] according to MMSE. It has been suggested that gingival inflammation, which is a result of prolonged oral hygiene deterioration, increases as cognitive impairment worsens [39]. ODB was not associated with nutrition according to MNA but eating soft or pureed food was associated with increasing ODB.

In this study the only significant general health condition associated with ODB was coronary artery disease (CAD), but ODB was not associated with dementia or diabetes. The load of multi-morbidity may mask the role of oral health in the overall load. Therefore, any associations between oral and general health may be difficult to identify because of the overwhelming burden of age- and disease-related changes [40].

ADS, which is a mathematical modelling of oral pathologies, has been modified from Total Dental Index measuring the severity of infections of the teeth and the periodontium both clinically and from radiographs [41]. The explanatory ability of ADS was validated by comparing it to that of the Total Dental Index, and it was originally used to examine whether the ADS was associated with known predictors of CAD [24]. We used ADS in our study because we find it useful in assessing the total disease burden of natural teeth affected by biofilm diseases. ADS (either as an original version including X-ray findings or as modified for the purposes of this study including clinical findings) combines the consequences of the most common diseases related to natural teeth. All of them are regarded as signs of bad oral health which may cause systemic problems mediated by bacteremia or through circulating inflammatory mediators. Our clinical ADS, which gives emphasis to teeth with particularly deep (≥ 6 mm) periodontal pockets [25] and from which the X-ray findings had been omitted, had a clear relationship with oral symptoms and signs which we tested as potentially easily detectable signs for nurses.

Several oral health assessments have been developed for use by non-dental healthcare professionals such as nurses and caregivers with multiple elements to be assessed both in the oral cavity and functionally [42]. Our goal was to find easily detectable surrogate markers that could be used to identify poor oral health, and further develop a screening test for this purpose. If the number of teeth were included in this diagnostic test especially aimed for nursing home staff, there would be room for interpretation of the test result, which would make its use in practical situations more complex. Our six signs selected for further testing (oral signs score-6) were linearly associated with the level of ODB according to ADS groups. However, all these signs used alone were weak to identify high ODB. Additive number of signs was associated with increasing ADS score level. When signs were observed as a combination of two or more, an excellent sensitivity value of 89% was achieved in detecting older adults with the highest ODB indicating that the test provided only a few false negative results, and true disease would not be overlooked. Thus, oral signs score-6 could be used as a screening tool for oral health problems in long-term care facilities. The purpose of a screening test is to lead to a more detailed oral examination by the dentist and an assessment of the need for treatment.

What matters is how caregivers recognize and interpret the oral symptoms and findings of the older adults. No sign alone is reliable, but as non-invasive methods they could be feasible in addition to other day-to-day care activities as means of determining whether a resident needs professional oral health care, i.e., to be evaluated by a dentist or oral hygienist. The individual signs of poor oral health found in this study could be considered as part of nursing home staff training. An intervention study among nursing home staff related to the use the signs score-6 items should be implemented.

Our results have been obtained from an average age of over 80 people living in long-term care facilities. Therefore, generalizability of the results and utilization of signs score-6 to other age groups and different living conditions need to be further investigated.

## Conclusions and implications

In conclusion, increasing oral disease burden associated especially with functional decline. The only general health association with increasing ODB in our sample was a higher rate of CAD. Our modified clinical ADS score seemed to capture well the overall oral disease burden. Of the older adults who participated in this study 35% had high ODB. We detected 6 easily detectable signs which could be used by nursing home staff to identify older adults with severe oral problems if 2 signs occur at the same time. As a practical implication, signs identified in this study may potentially be used as a screening test to identify a dentate older adult with oral problems.

## Data Availability

The data that support the findings of this study are available from the City of Helsinki but restrictions apply to the availability of these data, which were used under license for the current study, and so are not publicly available. Data are however available from the authors upon reasonable request and with permission of the City of Helsinki.

## Abbreviations

FINORAL: The Finnish Oral health studies in Older Adults
ODB: Oral disease burden
ADS: Asymptotic dental score
MMSE: Mini-mental state examination
CDR: Clinical Dementia Rating
MNA: Mini Nutritional Assessment
BMI: Body mass index
PI: Plaque index
GI: Gingival index
PPD: Pocket probing depth
BOP: Bleeding on probing
SD: Standard deviation
CI: Confidence interval
CAD: Coronary artery disease
ROC: Receiver operating characteristic
AUC: Area under the receiver operating curve
